# Computed Tomography–Based Differentiation of Benign and Malignant Craniofacial Lesions in Neurofibromatosis Type I Patients: A Machine Learning Approach

**DOI:** 10.3389/fonc.2020.01192

**Published:** 2020-07-31

**Authors:** Cheng-Jiang Wei, Cheng Yan, Yan Tang, Wei Wang, Yi-Hui Gu, Jie-Yi Ren, Xi-Wei Cui, Xiang Lian, Jin Liu, Hui-Jing Wang, Bin Gu, Tao Zan, Qing-Feng Li, Zhi-Chao Wang

**Affiliations:** ^1^Department of Plastic and Reconstructive Surgery, Shanghai Ninth People's Hospital, Shanghai Jiao Tong University School of Medicine, Shanghai, China; ^2^Department of Radiology, Zhongshan Hospital, Fudan University, Shanghai, China; ^3^Shanghai Institute of Medical Imaging, Fudan University, Shanghai, China; ^4^Department of Medical Imaging, Shanghai Medical College, Fudan University, Shanghai, China; ^5^Department of Radiology, Shanghai Ninth People's Hospital, Shanghai Jiao Tong University School of Medicine, Shanghai, China

**Keywords:** computed tomography, craniofacial lesion, neurofibromatosis type I, malignant peripheral nerve sheath tumor, machine learning

## Abstract

**Background:** Because neurofibromatosis type I (NF1) is a cancer predisposition disease, it is important to distinguish between benign and malignant lesions, especially in the craniofacial area.

**Purpose:** The purpose of this study is to improve effectiveness in the diagnostic performance in discriminating malignant from benign craniofacial lesions based on computed tomography (CT) using a Keras-based machine-learning model.

**Methods:** The Keras-based machine learning technique, a neural network package in the Python language, was used to train the diagnostic model on CT datasets. Fifty NF1 patients with benign craniofacial neurofibromas and six NF1 patients with malignant peripheral nerve sheath tumors (MPNSTs) were selected as the training set. Three validation cohorts were used: validation cohort 1 (random selection of 90% of the patients in the training cohort), validation cohort 2 (an independent cohort of 9 NF1 patients with benign craniofacial neurofibromas and 11 NF1 patients with MPNST), and validation cohort 3 (eight NF1 patients with MPNST, not restricted to the craniofacial area). Sensitivity and specificity were tested using validation cohorts 1 and 2, and generalizability was evaluated using validation cohort 3.

**Results:** A total of 59 NF1 patients with benign neurofibroma and 23 NF1 patients with MPNST were included. A Keras-based machine-learning model was successfully established using the training cohort. The accuracy was 96.99 and 100% in validation cohorts 1 and 2, respectively, discriminating NF1-related benign and malignant craniofacial lesions. However, the accuracy of this model was significantly reduced to 51.72% in the identification of MPNSTs in different body regions.

**Conclusion:** The Keras-based machine learning technique showed the potential of robust diagnostic performance in the differentiation of craniofacial MPNSTs and benign neurofibromas in NF1 patients using CT images. However, the model has limited generalizability when applied to other body areas. With more clinical data accumulating in the model, this system may support clinical doctors in the primary screening of true MPNSTs from benign lesions in NF1 patients.

## Introduction

Neurofibromatosis type 1 (NF1) is a common autosomal dominant genetic disorder with an incidence of ~1 in 3,000 individuals worldwide (1). The majority of affected individuals are predisposed to benign peripheral nerve sheath tumors (PNSTs), including cutaneous neurofibromas and plexiform neurofibromas (PNFs) (2, 3). Up to 10% of cases suffer from disfigurement and dysfunction caused by craniofacial lesions and often undergo plastic surgeries (4). As it is a benign tumor, most patients experience a course of treatment for several years without worrying about malignant transformation or tumor metastasis. However, 5–10% of PNF patients could develop NF1-related malignant peripheral nerve sheath tumors (MPNSTs) (5). Malignant peripheral nerve sheath tumor is an aggressive peripheral nerve tumor and is the leading cause of death in these patients, with a 50% rate of metastasis at the time of presentation and a dismal prognosis of several months (6, 7). Therefore, early diagnosis and intervention are vital, and complete surgical resection, if possible, improves prognosis (8, 9).

Radiology plays a vital role in the diagnosis and management of patients with NF1. Several imaging modalities or imaging-dependent methods, including magnetic resonance imaging (MRI) (10), fluorodeoxyglucose (FDG) positron emission tomography (PET), and PET/computed tomography (CT) (11), and PET/CT-guided percutaneous biopsies (12), are considered to be essential in determining malignancy formation, especially MPNSTs arising from benign PNFs in NF1 patients. Clinical studies even recommended NF1 adult patients to conduct whole-body MRI as routine medical surveillance (13). However, the methods mentioned above are costly; thus, there is an urgent and continued need for more economical imaging methods for the preliminary evaluation of MPNSTs in NF1 patients.

Computed tomography provides a safe and relatively affordable method for first evaluating head and neck NF1, but this anatomic imaging method was not effective enough to distinguish MPNST from benign NF1 (14). However, the development of a machine learning system might provide another way to obtain information from CT images. In this work, we explore a robust pattern recognition method based on Keras neural networks for differentiating between benign NF1 and MPNST with CT images (15).

## Materials and Methods

The data obtained for this analysis were approved by the local institutional review board (approval no. SH9H-2019-T163-2), and the authors had control of these data.

### CT Image Data Acquisition

We retrospectively reviewed the cases of 50 patients with benign plexiform neurofibromatosis type 1 (PNF) in the head and neck. Consistent with this, six patients with NF1-related head and neck MPNST were also included. All patients underwent contrast CT scan, and a total of 133 benign PNF slices and 33 MPNST slices were selected as training set. Furthermore, 9 other benign PNF and 11 MPNST slices from the CT images of these patients made up the new testing set. All these images were collected from the image archive and communication system in Shanghai Ninth People's Hospital between December 2012 and April 2019. The selections of slices were carried out by two experienced radiologists and an experienced doctor whose expertise was in head and neck NF1. They carefully chose slices with typical tumor region included. Meanwhile, another criterion for selections was that slices from each patient should be no more than 10 slices. Unlike in other tumors, there is no approved radiography guideline for the differentiation of benign and malignant NF1. As a result, no feature selection or classification before training could be carried out. To minimize the influences of non-tumor parts in the image, most images were selected at a similar slice of the head based on the typical tumor regions included. The images were collected in DICOM format, and the slice thickness was 5 mm.

**Figure 1:**
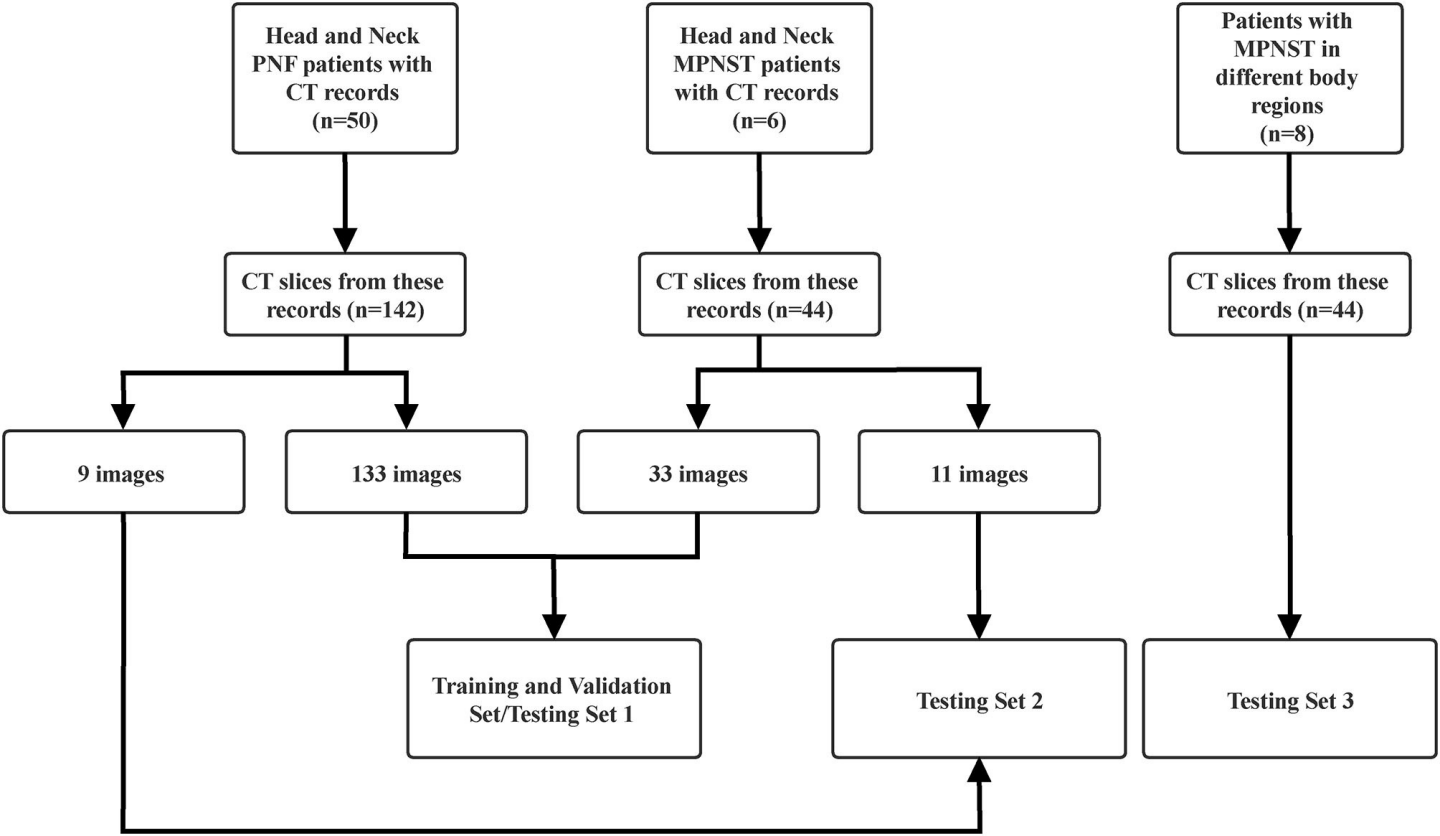
Flowchart shows the constitution of each set.

To examine the possible usage of this model in diagnosing MPNST from benign NF1 in different body regions beyond the head and neck, we collected images from eight MPNST patients in another hospital with different tumor locations, including the waist, mediastinum, legs, liver, heart, chest walls, retroperitoneum, and head. Twenty-nine slices were selected from these images to build the third testing set. The constitution of each set is shown in Figure 1. Matplotlib, a Python package, was applied to read and resize the images to standardize them to the same size. All the diagnoses had been previously proven by pathological biopsy.

### Keras Machine-Learning Model

The machine learning techniques comprised 2 steps. The first was a training step that used the CT images and an input–output system to train the model. This step was followed by the testing step, where the prediction model was tested for accuracy. Keras, written in Python language and working on top of TensorFlow, CNTK, or Theano, is a high-level neural network application programming interface (15). In general, Keras, as a neural network learning package of biological networks, contains an input layer, one or several hidden layers, and an output layer.

**Figure 2:**
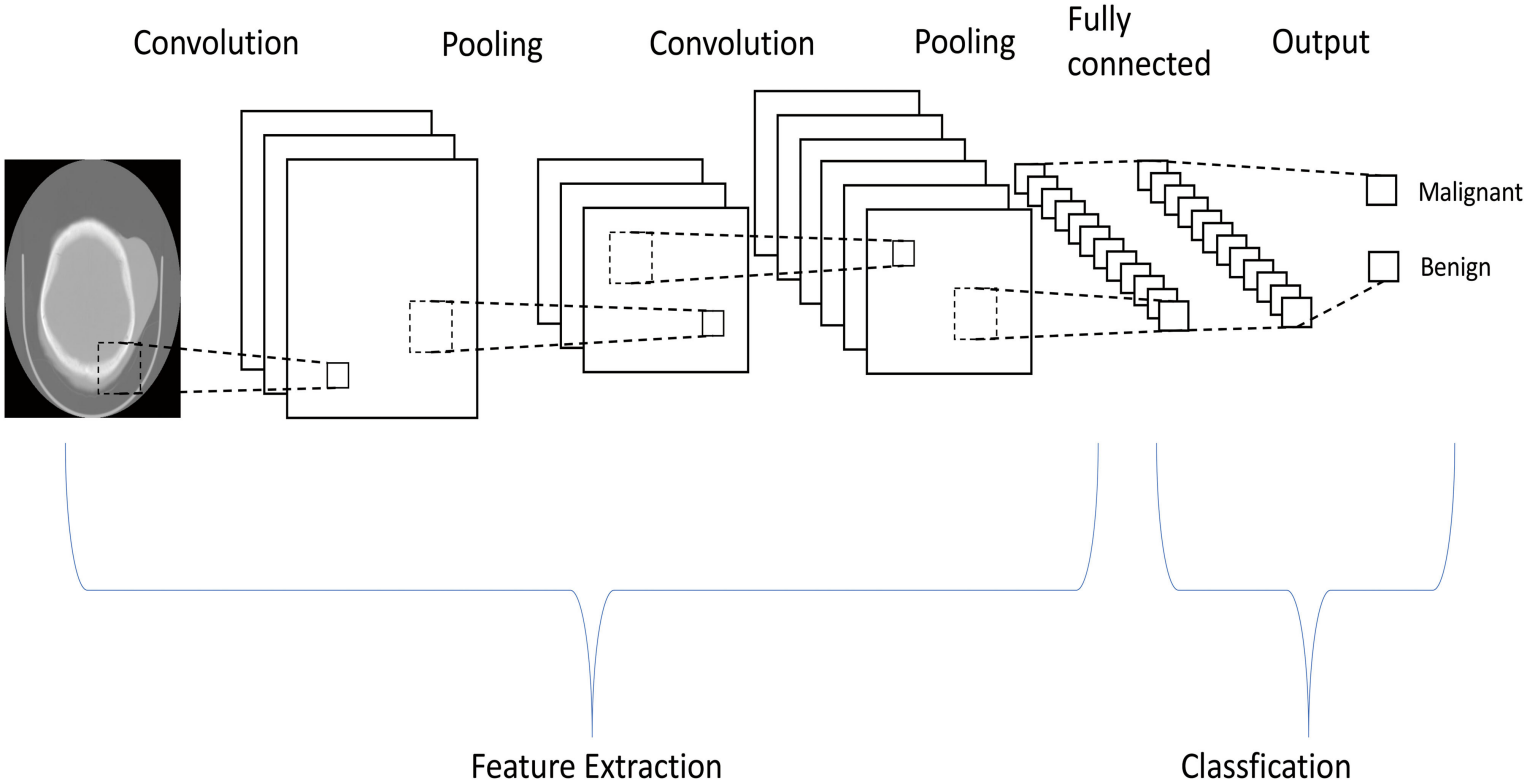
The machine learning system uses pooling layers generated from convolution layers multiple times to reduce the features in the computed tomography images, and only critical parts are retained.

**Figure 3:**
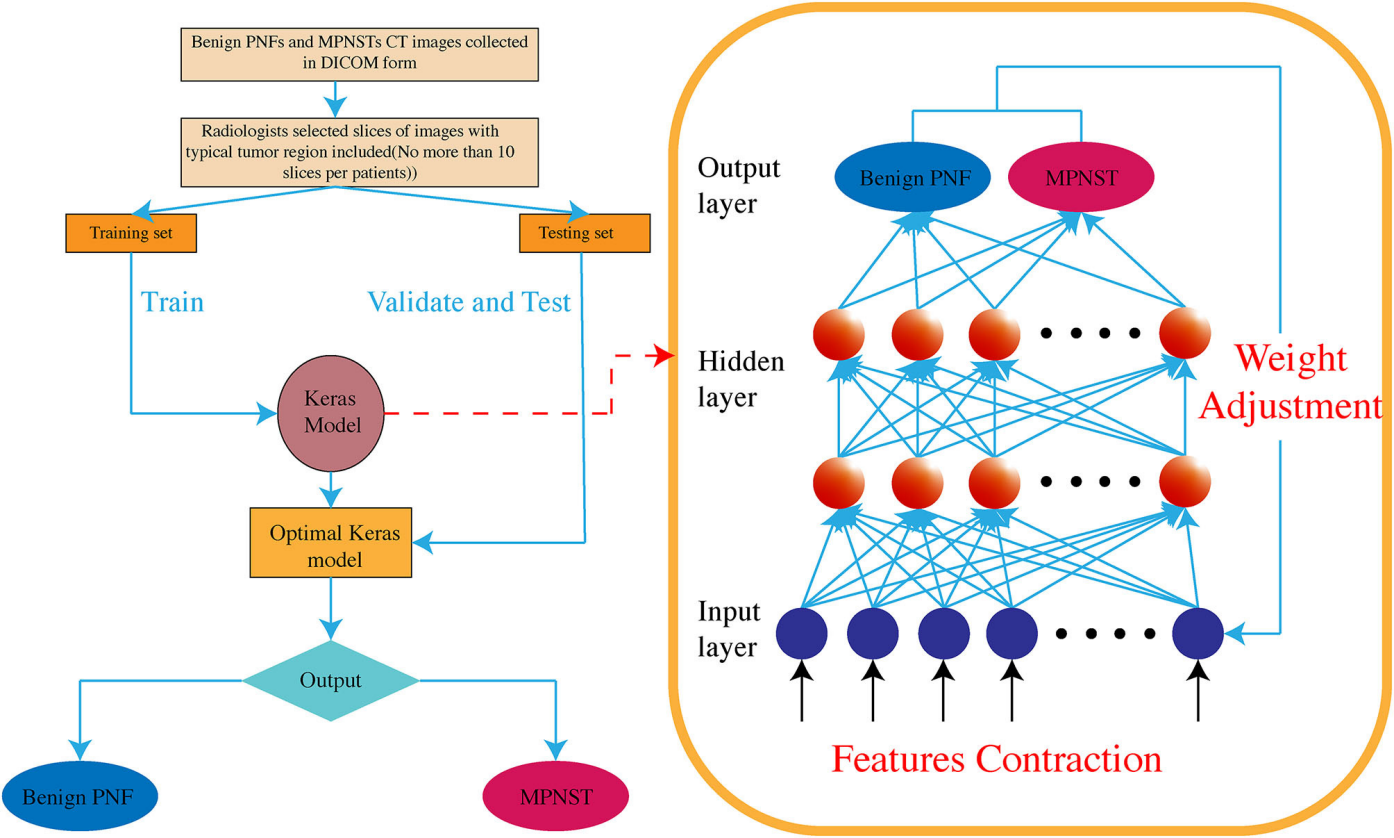
The Keras-based machine learning system is a high-level neural network, containing multiple hidden layers between input and output layers. Non-linear relationships are established among these hidden layers.

In this work, we used the Keras and TensorFlow framework to build the model for differentiating benign NF1 and MPNST. Supervised learning was applied in the model training. First, convolution layers were applied to derive the features of each image by sliding kernels (size = 3) through convolution. Then, pooling layers generated from the convolution layers were used to reduce the features and retain the most critical parts (Figure 2). Each image model exhibited two non-linear relationships between the input and output layers (Figure 3). For imbalanced number between benign PNF slices and MPNST slices, we adopted oversampling for relatively final balanced training cohort. All the images were tested multiple times, and the machine calculated the classification rates of each image based on the image characteristics explored by the machine. If the rate was more than 0.5, this image was assumed to be MPNST. Otherwise, it would be recognized as benign NF1. A rate closer to 0.5 showed that the machine was vacillating about the malignancy, whereas the opposite indicated a firm belief in the identification. The program codes for building, training, and validating this model are shown in Supplemental Materials 1, 2.

## Results

### Model Training and Validation

An entire machine learning system is mainly composed of three data sets: a training set, a validation set that generally arises from the training set by the machine automatically, and a testing set. The training set comprised a total of 133 benign head and neck NF1 and 33 MPNST images, and the machine randomly chose 90% of the training set to build the validation set. The characteristics of the study participants are summarized in Table 1. We first built a relatively simple model with less density to prove the practicability of the Keras-based machine learning method in performing the differentiation. After 16 epochs of training, the total accuracy of the training set was 83.33%. The model was then applied in the validation set, and the validation accuracy was 77.78%. This model indicated the diagnostic ability of the Keras-based system, but the accuracies needed further improvement.

**Table 1:** Patients characteristics.

**Characteristic**	**Training and validation set/testing set 1** **(*n* = 56)**	**Testing set 2** **(*n* = 6)**	**Testing set 3** **(*n* = 8)**
Age^*^	19 (10–25)	16 (14–45)	55 (48–70)
Male	27	4	7
MPNST patients	6	3	11

*Unless otherwise specified, data are number of patients*.

* *Data are medians, with interquartile ranges in parentheses*.

As the numbers of benign NF1 and MPNST images were different, a condition called imbalanced data, we used the auto imbalanced function of Keras to create a balanced batch generator to train this model. Then, we applied 24 epochs of training, and the total accuracy of the training set was 90.74% in this new model, whereas the validation set accuracy reached 88.89%. Both of these results indicated the high performance of this Keras-based machine-learning model in differentiating MPNST and benign neurofibroma by CT images.

### Model Testing

For the relatively rare images from MPNST patients, we first had to use the same image samples that we used to train the model to build the first testing set, and the total accuracy was 96.99%. The total trainable parameters were 32,865, and the parameters in each layer are listed in Table 2. However, presenting the same testing data is commonly believed to increase the accuracy beyond the real ability of the machine-learning model. To make this model more convincing, we then built another testing set composed of novel images, selecting 9 other benign NF1 and 11 MPNST images. All these images were from the slices of images that were not chosen in the first training and testing sets, and all these slices also presented evident tumor regions. Images in this new testing set contained manifold types of non-tumor “backgrounds.” Surprisingly, the results were all unanimous with the pathological report, and the rates of each image are shown in Supplementary Table 1. These data of probabilities showed that this model could better diagnose benign NF1, and the variation of rates in each MPNST image of this model showed difficulties in verifying the malignant tumor. This phenomenon might result from the scarcity of training images of MPNST patients, which makes it difficult to explore all the characteristics of the model. With more images collected and training this machine-learning model, the accuracy will increase and show a better robust diagnostic performance.

**Table 2:** The process of convolution and pooling layers.

**Layer (type)**	**Output shape**	**Param #**
conv2d_4 (Conv2D)	(None, 64, 64, 32)	896
max_pooling2d_3 (MaxPooling2)	(None, 32, 32, 32)	0
conv2d_5 (Conv2D)	(None, 16, 16, 32)	9,248
max_pooling2d_4 (MaxPooling2)	(None, 8, 8, 32)	0
conv2d_6 (Conv2D)	(None, 4, 4, 64)	18,496
global_average_pooling2d_2 (MaxPooling2)	(None, 64)	0
dropout_3 (Dropout)	(None, 64)	0
dense_3 (Dense)	(None, 64)	4,160
dropout_4 (Dropout)	(None, 64)	0
dense_4 (Dense)	(None, 1)	65

In concert with this, the third testing set was also applied to this model to examine the possibility of its usage in tumors in other regions. The total accuracy was 51.72%, and the rates of each image are shown in Supplementary Table 2. The unsatisfactory results compared with the other 2 testing sets above suggested the limited usage of this machine-learning model only in differentiating head and neck benign NF1 and MPNST. Interference by non-tumor regions might be responsible for these results because the model might be confused by different “backgrounds.”

## Discussion

Abrupt pain, enlargement, and new neurological signs appearing in a short time are considered phenomena associated with the transformation from benign NF1 to MPNST (16). Malignant peripheral nerve sheath tumors often metastasize to other places, such as the brain, lung, liver, bone, and skin, and have a poor prognosis (9, 17). As a result, whole-body imaging is an efficient method for MPNST detection in NF1 patients (13). Whole-body MRI with diffusion-weighted imaging/apparent diffusion coefficient (ADC) was studied as a useful tool for NF1 patients who have a risk of transforming to MPNST (18, 19). The ADC value has been widely used as a marker in soft tissue imaging, and its usage in MRI could represent cellularity in tissues and signify malignancy (18, 20). Consistent with this, a study demonstrated serial whole-body ^18^F-FDG-PET/CT as an efficient screening tool for the detection of early-stage MPNST in NF1 patients (21). Additionally, these whole-body imaging tools present doctors with information for surgeries.

However, both of these methods either are very expensive or increase patient anxiety, and they require a relatively long time to obtain results. Compared with PET CT and whole-body MRI, CT images provided a relatively economical choice for NF1 patients. More importantly, most of the NF1 patients only have benign tumors with relatively long overall survival, which indicates that regular clinical follow-ups are required for these individuals. The CT, rather than PET/CT or whole-body MRI, is the most suitable tool for regular assessment. For first-time clinical assessment or if there is a need to find all tumor regions in the whole body, PET/CT and whole-body MRI are better choices. But for lifelong regular test, CT is a more affordable and widespread method.

Nevertheless, it has been well-established that no diagnostic CT feature has been confirmed in differentiating MPNST from benign neurofibroma (14). Our study evaluated a Keras-based machine-learning model to predict the malignancy of head and neck NF1 on CT images, which yielded a high and stable measure. This model using CT images based on machine learning technology provides a new tool for MPNST screening in NF1 patients and reduces the cost. Furthermore, CT is a routine test for most follow-up benign PNF patients, and the usage of CT images in first screening will significantly assist the early diagnosis of MPNST and finally contribute to early treatment. Patients with MPNST suspension by this model indicates further needs of biopsy or other clinical tests for confirmed diagnosis, and we also recommended these patients receiving more regular clinical follow-up.

The unsatisfactory results of the third testing set suggest the interference of non-tumor background. To reduce the impacts of these backgrounds, scientists usually apply large quantities of image data and draw a contour line to restrict the tumor regions to minimize the effects (22). Restricted by the limitation of the number of patients, the work could hardly be carried out in whole-body machine-learning model training. Nevertheless, given the exciting accuracies of the existing model in head and neck malignancy differentiation, the potential usage of machine-learning models in the future diagnosis of MPNST from benign NF1 in different regions should be seriously considered with more data added.

Our study is not devoid of limitations. First, because of the limited number of patients with head and neck MPNSTs, only a comparably small number of patients were included. Consistent with this, some patients with MPNST had no CT record in our hospital. Both of these reasons limit the performance of the Keras-based machine learning technique that relies on large datasets. For this reason, we first had to use the same training and test set, which might contribute to higher accuracies than in the real world. In concert with this, for relatively balanced training set, the usage of oversampling would also contribute to somewhat overfitting. Moreover, the result might be influenced by tumor heterogeneity because of the use of multiple images of one patient. Furthermore, there are no guidelines in radiography for MPNST compared with benign NF1 in the head and neck, limiting the usage of tumor features to preprocess the images. We could only choose to use similar slices to train the model to minimize the effects of non-tumor parts, which also restricts the selection of images in each CT set. However, we then used 9 new benign PNF images and 11 new MPNST images from the same patients but different slices of the image set to build another test set for the machine-learning model. Surprisingly, all the images in the new test set were correctly classified. The high accuracy indicates the future clinical usage of this model, at least as a useful primary screening tool for differentiating head and neck MPNST from benign NF1. Potentially, with extensive radiomics analyses and more data collected in the future, the accuracy of the machine-learning model could increase, and the model performance could be improved.

Machine learning technologies have been used in the medical field, especially in radiography. Their ability to differentiate benign nodules and malignant tumors by multiple types of imaging methods has been proven in thyroid nodules (23), lung cancers (24), and breast cancer (25). Beyond this, machine learning systems in these cancers have also been developed for recurrence prediction (26), therapy efficiency assessment (27), and tumor staging (28). Various types of machine learning tools were found usable in radiography, such as the extreme learning machines, support vector machines, and neural networks (29). However, the establishment of machine-learning models in NF1 is rare until now, and the restrictions of development are mainly two parts. First, the number of patients is relatively low, and the acquisition of images is much more difficult. In concert with this, no approved staging consensus has been made in NF1 radiography with circumstances. Our work indicates the potential future usage of machine learning in NF1, and more machine learning systems could be further explored by aided clinical doctors. Meanwhile, with the gradual complementarity of studies and consensuses in NF1, these models might show higher performance in more fields.

## Data Availability Statement

The datasets generated for this study are available on request to the corresponding author.

## Ethics Statement

The studies involving human participants were reviewed and approved by the Project of Biobank (NO. YBKA201901) from Shanghai Ninth People's Hospital, Shanghai Jiao Tong University School of Medicine. Written informed consent from the participants' legal guardian/next of kin was not required to participate in this study in accordance with the national legislation and the institutional requirements.

## Author Contributions

C-JW, CY, YT, Q-FL, and Z-CW: conception and design. BG, TZ, Q-FL, and Z-CW: administrative support. C-JW, CY, YT, WW, Y-HG, and J-YR: provision of study materials or patients. C-JW, CY, YT, X-WC, JL, XL, and H-JW: collection and assembly of data. C-JW, CY, YT, and Z-CW: data analysis and interpretation. All authors: manuscript writing and final approval of manuscript.

## Conflict of Interest

The authors declare that the research was conducted in the absence of any commercial or financial relationships that could be construed as a potential conflict of interest.
