# Supplementary material for: Computed Tomography–Based Differentiation of Benign and Malignant Craniofacial Lesions in Neurofibromatosis Type I Patients: A Machine Learning Approach
Source: Front Oncol. 2020 Jul 31;10:1192. doi: 10.3389/fonc.2020.01192 (PMC7411852; doi:10.3389/fonc.2020.01192)
Supplement: Supplementary file 1 [file Data_Sheet_1.docx]

Supplementary Table 1: The rates of each image in validation set 2

|  | MPNST | Benign NF1 |
| --- | --- | --- |
| Total rates | 1 | 1 |
| Image 1 |  | 0.0000000e+00 |
| Image 2 |  | 0.0000000e+00 |
| Image 3 |  | 0.0000000e+00 |
| Image 4 |  | 0.0000000e+00 |
| Image 5 |  | 0.0000000e+00 |
| Image 6 |  | 3.6287904e-03 |
| Image 7 |  | 2.0612180e-03 |
| Image 8 |  | 1.8775463e-05 |
| Image 9 |  | 1.4892225e-05 |
| Image 10 | 0.9181279 |  |
| Image 11 | 0.8128742 |  |
| Image 12 | 0.8382788 |  |
| Image 13 | 0.92790604 |  |
| Image 14 | 0.83381677 |  |
| Image 15 | 0.64991486 |  |
| Image 16 | 0.52694315 |  |
| Image 17 | 0.71550316 |  |
| Image 18 | 0.5585585 |  |
| Image 19 | 0.86331815 |  |
| Image 20 | 0.82330420 |  |

Supplementary Table 2: The rates and the diagnosis result of each image of validation set 3 by this model.

| Images | Rates | Benign (-) or malignant (+) |
| --- | --- | --- |
| Total | 0.5172413793103449 | / |
| Image 1 | 4.1723251e-07 | - |
| Image 2 | 2.0861626e-07 | - |
| Image 3 | 9.5138454e-01 | + |
| Image 4 | 0.0000000e+00 | - |
| Image 5 | 9.5659173e-01 | + |
| Image 6 | 9.4998217e-01 | + |
| Image 7 | 0.0000000e+00 | - |
| Image 8 | 9.5109218e-01 | + |
| Image 9 | 9.5619625e-01 | + |
| Image 10 | 9.5757633e-01 | + |
| Image 11 | 0.0000000e+00 | - |
| Image 12 | 0.0000000e+00 | - |
| Image 13 | 9.1170454e-01 | + |
| Image 14 | 3.4570694e-06 | - |
| Image 15 | 9.1049004e-01 | + |
| Image 16 | 9.0265250e-01 | + |
| Image 17 | 0.0000000e+00 | - |
| Image 18 | 9.7647583e-01 | + |
| Image 19 | 0.0000000e+00 | - |
| Image 20 | 9.1141903e-01 | + |
| Image 21 | 8.9591134e-01 | + |
| Image 22 | 0.0000000e+00 | - |
| Image 23 | 4.5312881e-02 | - |
| Image 24 | 3.6680460e-01 | - |
| Image 25 | 5.6066796e-07 | - |
| Image 26 | 7.7706859e-02 |  |
| Image 27 | 8.8223726e-01 | + |
| Image 28 | 5.9958357e-01 | + |
| Image 29 | 6.0289288e-01 | + |
